# The Study of Morbid Anatomy

**Published:** 1911-09-02

**Authors:** 


### THE STUDY OF MORBID ANATOMY.

Roughly speaking, the study of the whole of the science of medicine, as opposed to the art of medicine, is pathology. Pathology is the study of the nature of disease. Every form of investigation, whether it deals with the body and its ills during life, or after death, may be said to be embraced by pathology. Investigation, however, may be said to be divided into two main branches. Firstly, investigation which deals entirely with the living—that is to say, the form of investigation which comprises efforts to ascertain during life the situation and the main characters of the morbid condition from which a patient is suffering. Secondly, investigation which, commencing with examination of the body after death, endeavours later to trace out the various influences which have combined to produce destruction of life. The first form of investigation is termed clinical or bedside diagnosis, but for bedside diagnosis to be intelligent and based upon clear mental pictures of the leading features of disease, it is necessary that much should be known of the appearances of disease after death.

While clinical diagnosis deals with the manifestations of disease as they occur during life, pathology, as has been mentioned, deals largely with the manifestations of disease after death. Speaking broadly, the manifestations of disease as seen after death may be said to indicate a struggle of the body against some influence which has had its end in destruction of the life of the body. The manifestations of this struggle may be confined to one organ or may be evident in many. In one person death may occur when the manifestations of disease are much more limited, or in a much earlier stage, than in another. The same disease may present after death a great number of appearances. It may be repeated that if clear ideas are to be held at the bedside of the morbid processes which may be taking place within the body, an extensive knowledge should be possessed of the varied appearances which may be seen after death.

### The Aim of Pathology.

Stress is laid upon this comparatively elementary view of one aspect of pathology because the great majority of those who enter the medical profession will probably not take much part in advancing the science of medicine. They will be called upon to practise the art of medicine. While the ultimate aim of pathology is to discover the cause of disease and as far as possible to banish disease by prevention, the ills of mankind at present are unfortunately abundantly with us. While these ills are present with us accurate diagnosis is necessary in order that alleviation may be given, or perhaps cure effected in the person attacked. And the more sound the knowledge of the naked-eye appearances of disease, the more likely is the one who practises medicine to render valuable aid. In these days, when nearly all pathologists are mainly concerned with tracing the causes of disease and also, it may be mentioned, with endeavours not only to prevent disease, but to find remedies for disease, there is a risk that the teaching of morbid anatomy may be overlooked. However this may be, it is well to bear in mind that in aiming at a high ideal the commonplaces of everyday life must not be forgotten.

### Lung Conditions in Children.

It may be added that in spite of the great number of years in which morbid anatomy largely occupied the attention of those engaged in the study of pathology, there are still some subjects upon which the knowledge of morbid anatomy is defective. The examination of school-children has brought one such subject to light. The profession as a whole seems to have very little idea as to whether chronic tuberculosis of the lungs is or is not common in children. Some medical men examining children find the disease to be very frequent, others to be very rare. There could scarcely be a better illustration for the need of association of clinical diagnosis with a knowledge of morbid anatomy. Either the man who finds tuberculosis of the lungs to be frequent, or the man who finds it to be infrequent, must be deficient in some kind of knowledge. One evidently must find what is not present, or the other must be incapable of detecting what is present. In reality there can be no doubt that the former class of medical man is mainly in the wrong. His want of knowledge of morbid anatomy of disease of the lungs in children leads him to imagine that trifling abnormal physical signs are the evidence of grave disease. His want of knowledge may mislead him in more than one way. There are other forms of chronic disease of the lung which in children are more common than chronic tuberculous disease of the lung, and, consequently, not only does he diagnose disease of the lung when no disease exists, but he mistakes other forms of disease for tuberculosis.

### Appendicitis.

The history of appendicitis affords another illustration of the need for knowledge of morbid anatomy. Although the great Dr. Addison long ago recognised the appendix as the seat of origin of serious and fatal inflammation, and that after his time those working in the post-mortem room of the hospital with which he was connected recognised the truth of his teaching, it has only been in comparatively recent years that widespread attention has been paid to this small portion of our intestines. We sometimes hear the statement made that appendicitis is increasing. That this is true may be doubted. Deaths formerly attributed to acute peritonitis, or, in old ordinary phraseology, to acute inflammation of the bowels, were almost invariably cases of appendicitis. The disease now appears to be more common because everyone is on the lookout for it. This widespread vigilance which is adopted with regard to appendicitis makes it appear to be more common than formerly even in hospitals, because on the first suspicion of its presence many such cases are now hurried off to these institutions.

### Its Morbid Anatomy.

The morbid anatomy of the disease appendicitis by no means ends with a knowledge that inflammation and various degrees of severity of inflammation originate in the appendix. There are many other details of knowledge concerning the appendix which are of great importance at the bedside—for example, the appendix is not always in its usual situation. It may be lying on the upper surface of the liver, in the middle line hidden behind the mass of small intestines, or it may be on the left instead of on the right side of the abdomen. Inflammation of the appendix in these abnormal situations would give rise to great difficulty of diagnosis. Such positions, however, for the appendix are rare, but there are other conditions associated with appendicitis which may mislead. An attack of appendicitis may give rise to an abscess over the right side of the liver or even in the liver itself. Such an abscess may be present and its origin in appendicitis be entirely unsuspected. Not only may this be the case, but the inflammation may spread through the diaphragm and give rise to trouble in the pleura or in the lung, which for a time, at least, may lead to the supposition that the patient is suffering primarily from disease within the chest.

### The Significance of Post-mortem Signs.

Again, disease of appendix affords evidence to the naked eye of attempts made by the body to resist the invasions of disease. If the inflammation spreads from the appendix rapidly throughout the cavity of the abdomen, death occurs from poisoning by the products of disease. Attempts are, therefore, made to confine the inflammation to a limited area. In these attempts the great omentum plays an important part. This fold of peritoneum much more readily forms adhesions than any other part of the serous membrane. In a way which at first sight seems almost to suggest that the omentum is endowed with the powers of a free agent, it finds its way to the diseased spots. Adhesions are formed around the pus-forming area, and happily in many instances the power for harm is localised and the life saved. The great omentum plays a part in many affections of the abdomen, but perhaps in none is there such frequent evidence of its power for good as in appendicitis. This very power for good may, however, occasionally result in serious after-effects. An omentum which has become attached to an inflamed spot may, at some later period, cause obstruction of a coil of intestine, and the obstruction, if unrelieved by surgical interference, will occasion death. Many points might be drawn attention to in connection with the great omentum. These few remarks, however, are merely made to suggest to the student the various features of interest and value which may be learnt by using his eyes in the post-mortem room. Yet it may be permissible to mention in this connection that the student should not only take every opportunity of learning by the use of his eyes, but he must guard against allowing his vision to be biassed by what he has read or heard. Text-books are very useful, but they are not infallible. One writer is influenced by the writings of another, and he may feel that should his experience not agree with the generally accepted opinion, his own experience may have been exceptional and that, consequently, his opinion, based upon his experience, may be wrong. Not only may this be the case, but it is to be feared that the way in which we look at any object with our eyes, or at any subject with our mental vision, is apt to be seriously influenced by what we have been taught. Respect for authority, but not blind worship of authority, should be the attitude of the student. Consideration of the great omentum suggested the foregoing remarks, though possibly other parts of the body would have formed a better basis for them. Yet the omentum may be used as an illustration of what is meant by these remarks. At one time considerable stress was laid upon thickening of the great omentum as an important feature in the diagnosis of tuberculous peritonitis. It is true that there is a liability of the great omentum to become extensively invaded by tubercle. In the same way the great omentum may become extensively affected by malignant growth. To expect, however, this thickening to be frequently present in marked degree would be a mistake. It would be more correct to say that such cases are the exception, not the rule. Mention is made of this because here again are features of morbid anatomy which may be detected clinically. A thickened great omentum can readily be detected during life, and stress is still often laid upon the importance of its presence in the diagnosis of tuberculous peritonitis. When present it has its value, but the appearances in the post-mortem room produced by tuberculous peritonitis are very varied, and the greater the knowledge of these various appearances the more easy will it be to diagnose the disease during life.

Many other illustrations might be given in which teaching upon morbid anatomy may in some details mislead the student unless he check the teaching by personal observation. Most students seem to hold erroneous views with regard to the nature of the disease of the mitral valve, following rheumatism, which occasions mitral regurgitation. The views held by students would seem to indicate that in this matter the teaching of the text-books is more correct than the teaching they receive at the bedside, though the text-books in this matter are not always very clear guides. But it is not desired to labour points such as these. The fullest use should be made of the teachings of the experience of others, but let it be checked by one's own. Unfortunately, the experience of us all is very limited. In fields of medicine and morbid anatomy, however great may be the experience, variations of disease hitherto unmet with by the observer are frequently appearing. In this lies one great interest of the profession of medicine. Without forgetting that the patient is a human being in need of sympathy and consideration, he may be approached with the feeling that this illness provides material for adding to knowledge. This applies to the living subject, and perhaps still more to the dead. Knowledge gained by examination of the dead is more certain than knowledge gained by examination of the living. It is the comparative definiteness of knowledge gained in the post-mortem room which makes it necessary for the student to store his mind well with its teaching during his hospital career. When once in practice his opportunities for testing the accuracy of his diagnosis will be comparatively rare.

A few words may be said with regard to microscopical morbid anatomy. As an aid to diagnosis in many affections the microscope is of great value, but not many medical men when in practice conduct such examination for themselves, with the exception of those examinations which are comparatively simply carried out. Yet the student will find his study of disease by the aid of the microscope of great value. It is the microscope which reveals something of the nature of disease, and shows how the naked-eye appearances are in the majority of instances the results of attempts of the body to resist some attacking force. In his study of these processes he will find not only much to interest, but much to stimulate thought. He will find, for example, in two conditions of consolidation of the lung that in one exudation from the blood forms the greater part of the inflammatory exudation, in another that the exudation is largely composed of cells derived from the lung itself. In the form in which the exudation is largely from the blood, the disease terminates suddenly by a definite crisis, and that although the consolidation of the lung for several days remains the same, the patient, after the crisis, is comfortable and out of danger. In the other form of consolidation, if the patient lives so long, the disease may last for several weeks, and if the disease should pass away it does so gradually. Consideration of points such as these will show him that, although knowledge of morbid anatomy is of great importance where diagnosis is concerned, naked-eye and microscopical appearances are only evidences of mysterious forces at work. At present little is known of these forces and of means to combat them. To combat them is the aim of medicine, but should the power for good of the science of medicine be greatly increased, accurate diagnosis and the knowledge of morbid anatomy, upon which it is based, will still be necessary.

